# Genomic, proteolytic, and phenotypic characterization of *Pseudomonas aeruginosa* isolates causing infective endocarditis

**DOI:** 10.1128/spectrum.03742-25

**Published:** 2026-05-12

**Authors:** Sarah M. Hickson, Von Vergel L. Torres, Jing Jie Teh, Ian O’Keefe, Robert K. Ernst, Mark Morrison, Kate L. McCarthy, Timothy J. Wells

**Affiliations:** 1 Frazer Institute, The University of Queensland 1974, Brisbane, Queensland, Australia; 2 Institute of Molecular Bioscience, The University of Queensland 1974, Brisbane, Queensland, Australia; 3 Centre for Microbiome Research, School of Biomedical Sciences, Queensland University of Technology (QUT), Translational Research Institute 373031 , Woolloongabba, Queensland, Australia; 4 Department of Microbial Pathogenesis, University of Maryland School of Dentistry 89015, Baltimore, Maryland, USA; 5 Department of Microbiology, Pathology Queensland, Brisbane, Queensland, Australia; 6 Royal Brisbane and Women’s Hospital, Brisbane, Queensland, Australia; 7 Australian Infectious Diseases Research Centre, The University of Queensland 1974, Brisbane, Queensland, Australia; Yan'an University, Yan'an, Shaanxi, China

**Keywords:** infective endocarditis, *Pseudomonas aeruginosa*, virulence factors, proteases, proteomics

## Abstract

Infective endocarditis (IE) remains a formidable clinical challenge, characterized by high morbidity and mortality despite advances in antimicrobial therapy and surgery. *Pseudomonas aeruginosa* is an increasingly relevant IE pathogen due to its intrinsic antibiotic resistance and rising incidence among patients with cardiac implantable electronic devices. However, the specific virulence factors that facilitate *P. aeruginosa* colonization and persistence in IE are poorly defined. In this study, we analyzed six *P. aeruginosa* strains isolated from IE patients and compared them genomically, proteomically, and phenotypically to a reference panel of 20 well-characterized clinical isolates from diverse *P. aeruginosa* infection types. All strains were genome sequenced for genetic comparison. The proteome of five IE isolates was analyzed by mass spectrometry and compared to the proteomes of *P. aeruginosa* reference strains (PAO1, PA14, and PA_O6) grown in similar conditions. Finally, the entire panel of isolates was analyzed for phenotypes, including motility, biofilm formation, protease activity, serum resistance, pyocyanin production, and cell lysis. While all strains exhibited high genomic similarity, IE isolates lacked a distinct genomic signature. Proteomic profiling revealed consistent upregulation of virulence-associated proteins in IE strains, including components of quorum sensing (e.g., *Pseudomonas* Quinolone Signal), proteases (e.g., elastase), adhesins (e.g., type IV pili), and toxins (e.g., phenazines), relative to non-IE strains. Phenotypic assays demonstrated that IE isolates displayed traits commonly linked to acute infections, such as O-antigen expression, serum resistance, enhanced motility, and hemolytic activity. Notably, IE strains exhibited significantly elevated proteolytic activity compared to most other clinical isolates, underscoring the potential role of proteases in the pathogenesis and persistence of *P. aeruginosa*-mediated infective endocarditis.

**IMPORTANCE:**

*Pseudomonas aeruginosa* infective endocarditis (IE) is a rare but life-threatening condition with limited understanding of its pathogenesis. This study presents the first comprehensive characterization of clinical isolates from patients with *P. aeruginosa* IE, integrating genomic, proteomic, and phenotypic analyses in comparison with reference strains and a diverse array of clinical isolates. Although no distinct molecular signature was identified, the IE isolates consistently displayed virulence traits and phenotypes commonly associated with acute infections. Proteomic profiling revealed increased expression of prominent virulence factors, including phenazines, proteases, and pili, that likely contribute to disease initiation and persistence. Notably, all IE isolates exhibited markedly elevated proteolytic activity, significantly higher than in isolates from other clinical contexts.

## INTRODUCTION

Infective endocarditis (IE) is a serious infection of the inner lining of the heart (endocardium). IE typically follows episodes of bacteremia, where circulating bacteria colonize damaged or abnormal cardiac tissue (1), often forming cellular aggregates called vegetations. These vegetations can obstruct blood flow, impair cardiac function, and increase the risk of systemic embolization. Individuals with implanted devices (e.g., pacemakers and artificial valves) and pre-existing cardiac conditions are particularly susceptible, as their altered endocardial surfaces provide a favorable environment for bacterial adherence and colonization (2). Cardiac damage can be generated from physical damage as a result of surgery (3), intravenous drug use (4), previous cases of IE (5, 6), and persistent bacteremia (7, 8).

Gram-positive bacteria, particularly *Staphylococcus*, *Streptococcus*, and *Enterococcus* species, dominate IE pathogenesis and have well-characterized virulence factors. In contrast, gram-negative pathogens are uncommon causes of IE, with species such as *Pseudomonas aeruginosa* historically accounting for less than 2% of cases (9). Although *P. aeruginosa* IE is uncommon, it carries a high mortality rate of approximately 26%, and more than one-third of patients experience relapse even after receiving appropriate therapy (9–12). The rate of complications, including mortality and infection relapse, has now exceeded that of *Staphylococcus aureus* IE (13). Over the past decades, the incidence of *P. aeruginosa* IE has been increasing (14, 15). Due to the increasing prevalence of *P. aeruginosa* IE, this bacterium was listed in the 2023 Duke Criteria as a “typical” pathogen associated with device-related IE (14).

Despite the rise in incidence, there remains very little known about the factors driving *P. aeruginosa* endocarditis pathogenesis. Notably, no publicly available genomes exist for *P. aeruginosa* isolates derived from endocarditis cases, highlighting a significant gap in genomic resources. Although extensive literature explores the virulence factors of *P. aeruginosa* in respiratory, bloodstream, and other infections, their role in IE remains almost entirely unexamined. Identifying potential genomic, phenotypic, or proteomic signatures unique to *P. aeruginosa* strains that cause IE can suggest bacterial factors important for *P. aeruginosa* pathogenesis in this disease. Identifying factors important for *P. aeruginosa* IE will allow targeted antimicrobial therapies, which may prevent the development of this devastating disease.

In this study, we characterize six clinical *P. aeruginosa* isolates from patients with infective endocarditis (hereafter referred to as IE isolates) in comparison to a panel of 20 clinical isolates from non-IE infections. Well-characterized reference strains PAO1, PA14, and PA_O6 are also included to provide context and standard comparisons. Reference strains selected are commonly used as laboratory strains, modeling moderate (PAO1) and hypervirulent (PA14) phenotypes, respectively, and serum sensitivity (PA_O6). This integrated omics approach seeks to differentiate IE isolates from other clinical strains or, alternatively, to assess whether *P. aeruginosa* strains possess a broad pathogenic potential that enables infection across diverse clinical presentations.

## RESULTS

### Genomic characterization of IE isolates

**Fig 1:**
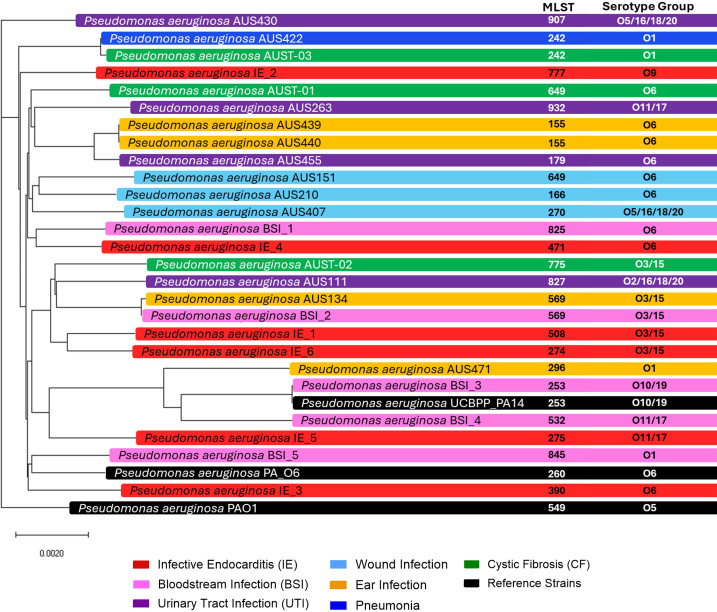
Phylogeny and classification of a panel of *P. aeruginosa* isolates from different infection types. Relatedness of *P. aeruginosa* isolates was visualized, and multilocus sequence type (MLST) and serotype were defined by genome sequence identity. Colors indicate infection source.

To evaluate genomic variation of IE isolates, *P. aeruginosa* was isolated from the blood of six patients with IE (12). A representative panel of 20 clinical strains from other infection types (urinary tract, wound, chronic lung, ear, and bloodstream) and reference strains, well-characterized *P. aeruginosa* strains (PAO1, PA14, and PA_O6), were selected for comparison. Whole-genome sequencing was performed on IE (*n* = 6) and bloodstream infection (BSI) isolates (*n* = 5). The genomes of the remaining panel isolates were publicly available. Genomic sequencing revealed that IE isolates were not clonal, shared no serotype group, nor multilocus sequence type (MLST), and demonstrated distal cladding (Fig. 1). Although no serotype was unanimous across IE isolates, O6 (2/6) and O3/15 (2/6) serotypes were most common.

**Fig 2:**
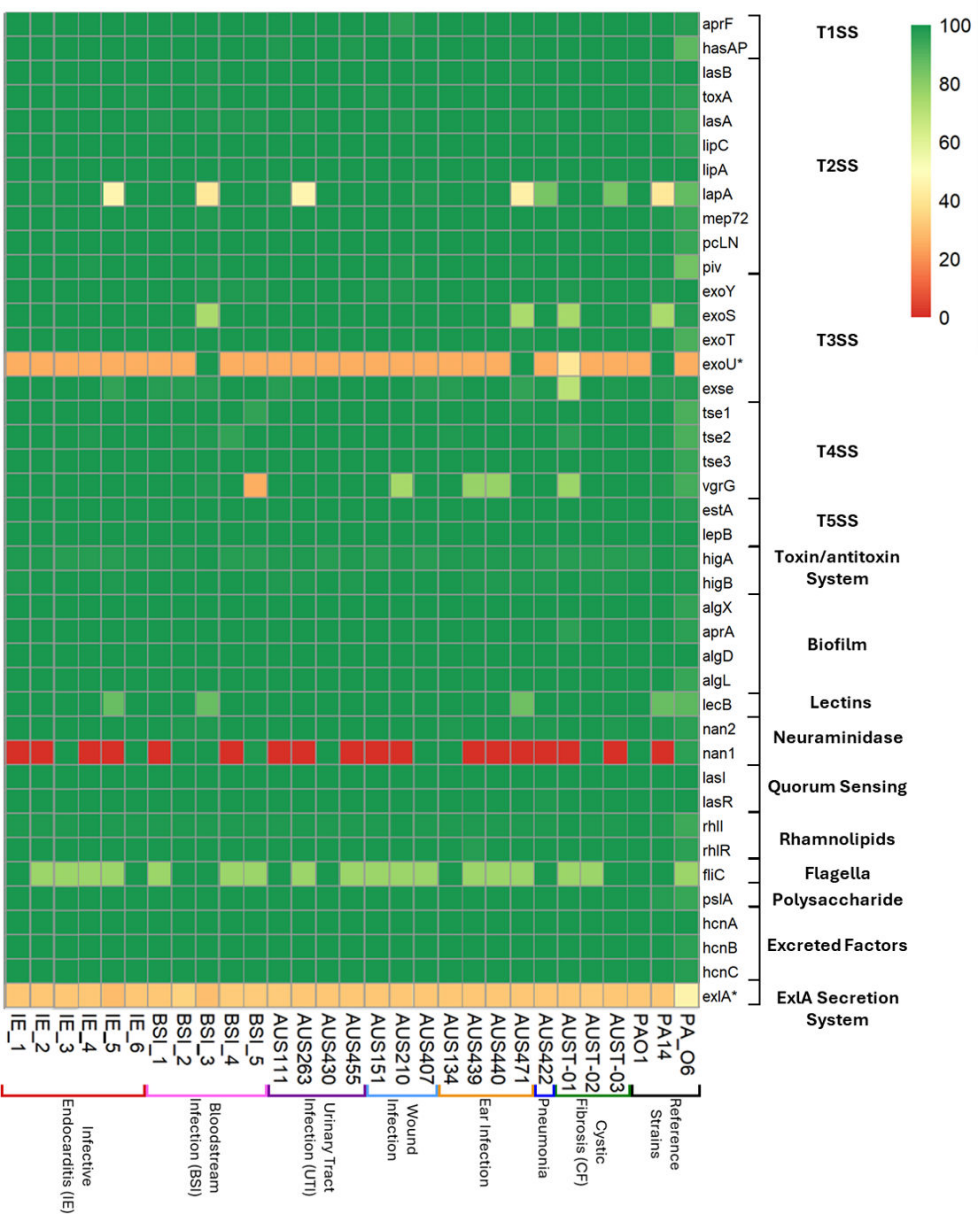
Virulence gene presence in a panel of *P. aeruginosa* isolates. BLAST similarity scores range from 100 (green) to 0 (red) percent for each virulence gene compared to gene sequences from PAO1. Genes absent from PAO1, and thus sequences taken from UCBPP-14 (PA14) (exoU) and PA7 (exlA), are indicated by an asterisk (*).

To investigate the virulence gene carriage of IE isolates, we screened for the presence or absence of a panel of known virulence genes previously investigated in *P. aeruginosa* bloodstream infection isolates (16) (Fig. 2) and detected antibiotic resistance genes (Table S1). All IE isolates possessed the vast majority of screened virulence genes, with 95% present in at least 1 IE genome and 86% present in all 6 IE genomes. The type 3 secretion system (T3SS) delivers cytotoxins via a needle-like structure, promoting host cell damage and *P. aeruginosa* virulence. Co-carriage of *exoU* and *exoS*, T3SS toxins, is rare in *P. aeruginosa* genomes (17). None of the IE isolates carried *exoU*, a gene linked to heightened virulence when compared to *exoS*-carrying strains (18). However, only three isolates of the entire panel carried *exoU*; thus, carriage of the cytotoxic gene is not significantly different from other isolates from different infection sources. Exolysin (*exlA*), a recently described bacterial toxin associated with hypervirulence (19), was not carried in any panel isolates’ genomes. Flagella are important bacterial components involved in motility, adhesion, and biofilm formation; however, two variants of this gene are described in *P. aeruginosa*. The majority of IE isolates encoded flagella type B (4/6), with the remaining encoding type A variant; however, all isolates do carry flagellin subunit protein *fliC*.

### Proteome characterization of IE isolates

**Fig 3:**
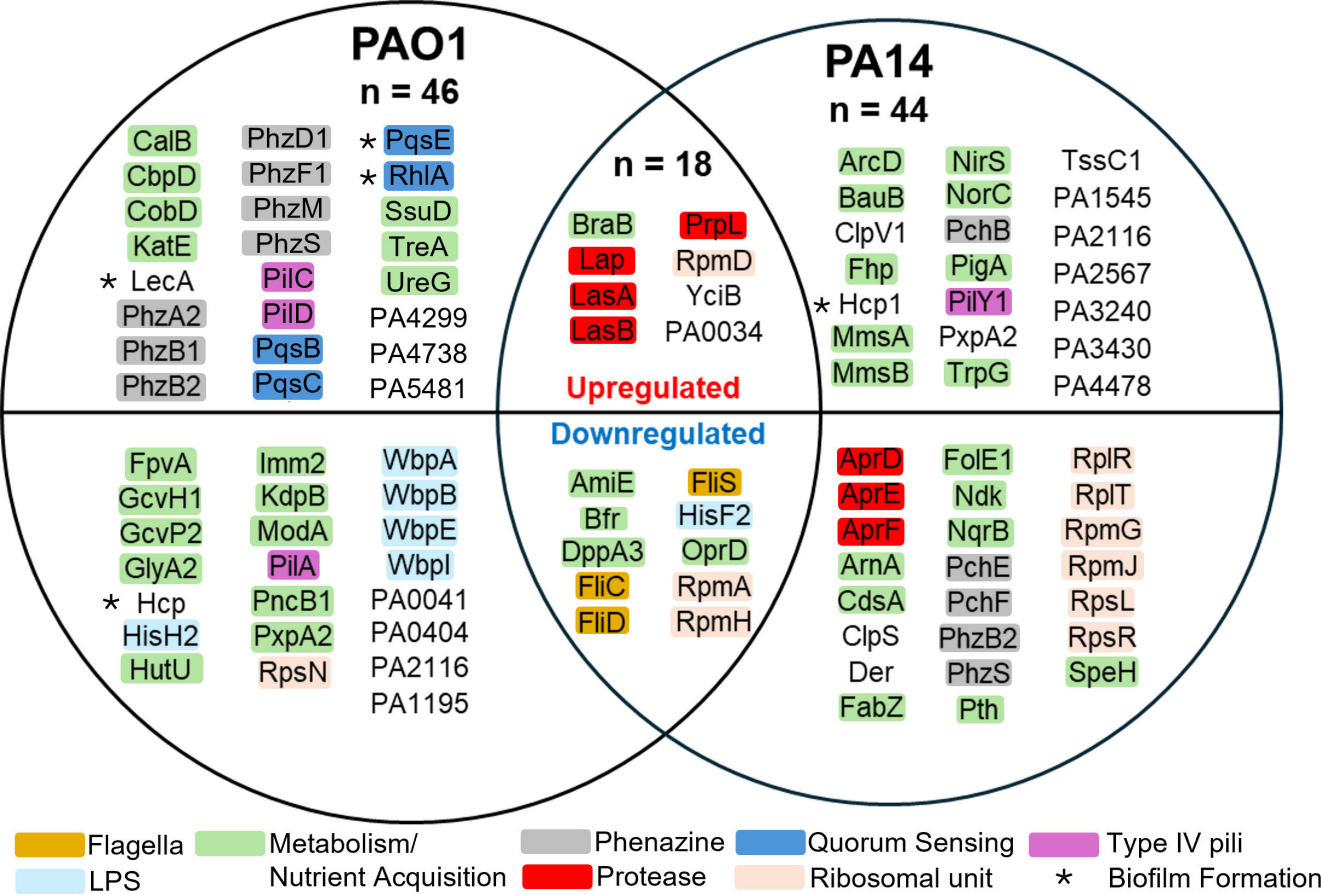
Proteins of IE isolates with significant changes in expression after growth in nutrient-rich media (lysogeny broth). The top half: significantly upregulated proteins in comparison to either/both PAO1 or PA14 expression. The bottom half: significantly downregulated proteins in comparison to either/both PAO1 or PA14 expression. Proteins listed in the intersection demonstrated significant (*P* < 0.05) expression differences in comparison to both PAO1 and PA14 in the majority of IE isolates (≥3). Genes are colored according to their predicted function.

Although IE isolates encoded many of the virulence genes, the expression of these factors can vary greatly between *P. aeruginosa* strains. To investigate bacterial expression, proteomes of IE isolates (*n* = 5) were compared to both reference strains PAO1 and PA14. Significant protein expression, in comparison to reference strains, was determined via greater than twofold change in abundance, <0.05 *P*-value, and was true for the majority of IE isolates (≥3). In total, 46 and 44 proteins were differentially expressed in the majority of IE isolates in comparison to the PAO1 and PA14 proteomes, respectively. Eighteen of these proteins were differentially expressed in IE isolates in comparison to both reference strains. Of these 18, 8 proteins were significantly upregulated, and 10 proteins were significantly downregulated in the majority of IE isolates (Fig. 3).

Proteins upregulated in IE isolates were associated with known virulence functions such as proteases, adhesion, and quorum sensing. Proteases are important for bacterial pathogenesis and virulence by degrading host proteins, aiding immune evasion, and pathogenicity (20, 21). Proteases upregulated in IE isolates included LasA and LasB involved in elastase (or pseudolysin) production, PrpL (or protease IV), a lysyl endopeptidase, and Lap involved in proteolysis with metalloexopeptidase activity. In comparison to PAO1, three proteins associated with alkaline protease (AprA) secretion were downregulated in the majority of IE isolates.

Adhesion is one of the initial steps in infection development. In *P*. aeruginosa, pili are surface appendages important for surface sensing, motility, and adhesion (22). Compared to the PAO1 proteome, two proteins (PilC and PilD) are upregulated in the majority of IE isolates, and both gene-coding regions are in the same operon. However, another protein associated with pili (PilA) was downregulated against PAO1, but this gene does not belong to the same operon. Compared to the PA14 proteome, one pili protein (PilY1) was upregulated in the majority of IE isolates. This protein sequence differs between PAO1 and PA14, and expression differences could be due to undetected variants or due to low expression under the tested conditions.

Quorum sensing is crucial for bacterial communication and is involved in regulating *P. aeruginosa* virulence, e.g., the transition from planktonic to sessile growth, relevant in biofilm formation (23). In comparison to PAO1, four key mediators of quorum sensing were upregulated in the majority of IE isolates. In comparison to PAO1, two *Pseudomonas* Quinolone Signal (PQS) involved in mediating quorum sensing (PqsB and PqsC) were upregulated in the majority of IE isolates. Expression of phenazines and LecA, an adhesive lectin associated with adherence to lung epithelial cells (24), is regulated by PQS (25). In comparison to PAO1, LecA and seven proteins associated with phenazine production were upregulated in IE isolates (PhzA2, PhzB12, PhzD1, PhzF, PhzF2, PhzM, and PhzS). In comparison to PA14, proteins regulated by quorum sensing involved in heme oxidation and heme-binding (PigA, Fhp, and NorC), or oxidoreductase activity (NqrB, MmsA, and MmsB), were significantly upregulated.

Overall, proteomic expression in IE isolates is suggestive of a virulent strain that can survive in diverse environments. In conjunction, factors associated with signaling and nutrient acquisition (e.g., phenazines), toxin secretion (e.g., proteases), and attachment (e.g., pili) are likely important for IE isolates’ virulence.

### IE antibiotic susceptibility

Antibiotic susceptibility was investigated via disk diffusion assay as per European Committee on Antimicrobial Susceptibility Testing (EUCAST) standards (26). Uniform susceptibility to antibiotics among IE isolates was only observed in response to amikacin (sensitive) and tobramycin (intermediate susceptibility; Table 1). The majority of IE isolates were susceptible-dose dependent (intermediate sensitivity) to antibiotics tested (Aztreonam, Amikacin, Ceftazidime, Cefepime, Ciprofloxacin, Meropenem, Tobramycin, and Piperacillin + Tazobactam). Isolates IE_4 and IE_6 demonstrated the highest resistance among IE isolates, reporting resistance to 4/8 and 6/8 antibiotics tested, respectively. Whereas IE_5 was resistant to 2/6 antibiotics, and the remaining isolates (IE_1, IE_2, and IE_3) were only sensitive or intermediately susceptible to the antibiotics tested. Antibiotic susceptibility trends of IE isolates were similar to other infection groups; however, cystic fibrosis (CF) isolates were resistant to the majority of antibiotics tested.

**TABLE 1:** Antibiotic sensitivity of the panel of *P. aeruginosa* isolates from different infection types^*a*^^,^^*b*^

	IE						BSI					UTI				Wound			Ear infection				P	CF			Reference			S
	IE_1	IE_2	IE_3	IE_4	IE_5	IE_6	BSI_1	BSI_2	BSI_3	BSI_4	BSI_5	AUS111	AUS263	AUS430	AUS455	AUS151	AUS210	AUS407	AUS134	AUS439	AUS440	AUS471	AUS422	AUST-01	AUST-02	AUST-03	PAO1	PA14	PA_O6	ATCC 27853
ATM30	I	I	I	R	I	R	I	I	I	R	R	I	I	I	I	I	I	I	I	I	R	I	I	I	I	R	I	I	I	I
AK30	S	S	S	S	S	S	S	S	S	R	S	S	S	S	S	R	S	S	S	R	S	S	S	R	S	R	S	S	S	S
CAZ10	I	I	I	R	I	R	I	I	I	R	I	I	I	I	I	I	I	I	I	I	I	I	I	I	R	R	I	I	I	I
CIP5	I	I	I	R	R	R	I	I	I	I	I	I	I	I	I	I	I	I	R	I	R	I	R	R	R	R	I	I	I	I
FEP30	S	S	S	R	R	R	S	I	S	R	S	S	S	S	I	S	S	S	S	I	I	S	S	R	R	R	S	S	S	S
MEM10	S	S	S	I	S	R	S	S	S	I	S	S	S	S	S	S	S	S	S	S	I	S	S	R	R	I	S	S	S	S
TOB10	I	I	I	I	I	I	I	I	I	R	I	I	I	I	I	I	I	I	I	R	I	I	I	R	R	I	I	I	I	I
TZP110	I	I	I	I	I	R	I	I	I	I	I	I	I	I	I	I	I	I	I	I	I	I	I	I	R	I	I	I	I	I

^*a*^ S, antibiotic sensitive; I, intermediately sensitive; and R, antibiotic resistance; IE, infective endocarditis; BSI, bloodstream infection; UTI, urinary tract infection; CF, cystic fibrosis; P, Pneumonia; ATM, Aztreonam; AK, Amikacin; CAZ, Ceftazidime; CIP, Ciprofloxacin; FEP, Cefepime; MEM, Meropenem; TOB, Tobramycin; and TZP, Piperacillin + tazobactam.

^*b*^ Highlighted cells are resistant to the antibiotics and shading is used to highlight these results.

### IE isolates have characteristics of acute *P. aeruginosa*

Bacterial factors important for IE development for other bacteria, such as *Staphylococcus* and *Streptococcus* species, include adhesion, persistence, cell/host toxicity, and translocation. Here, we determine the virulence phenotypes of *P. aeruginosa* IE strains compared to the panel of clinical strains.

**Fig 4:**
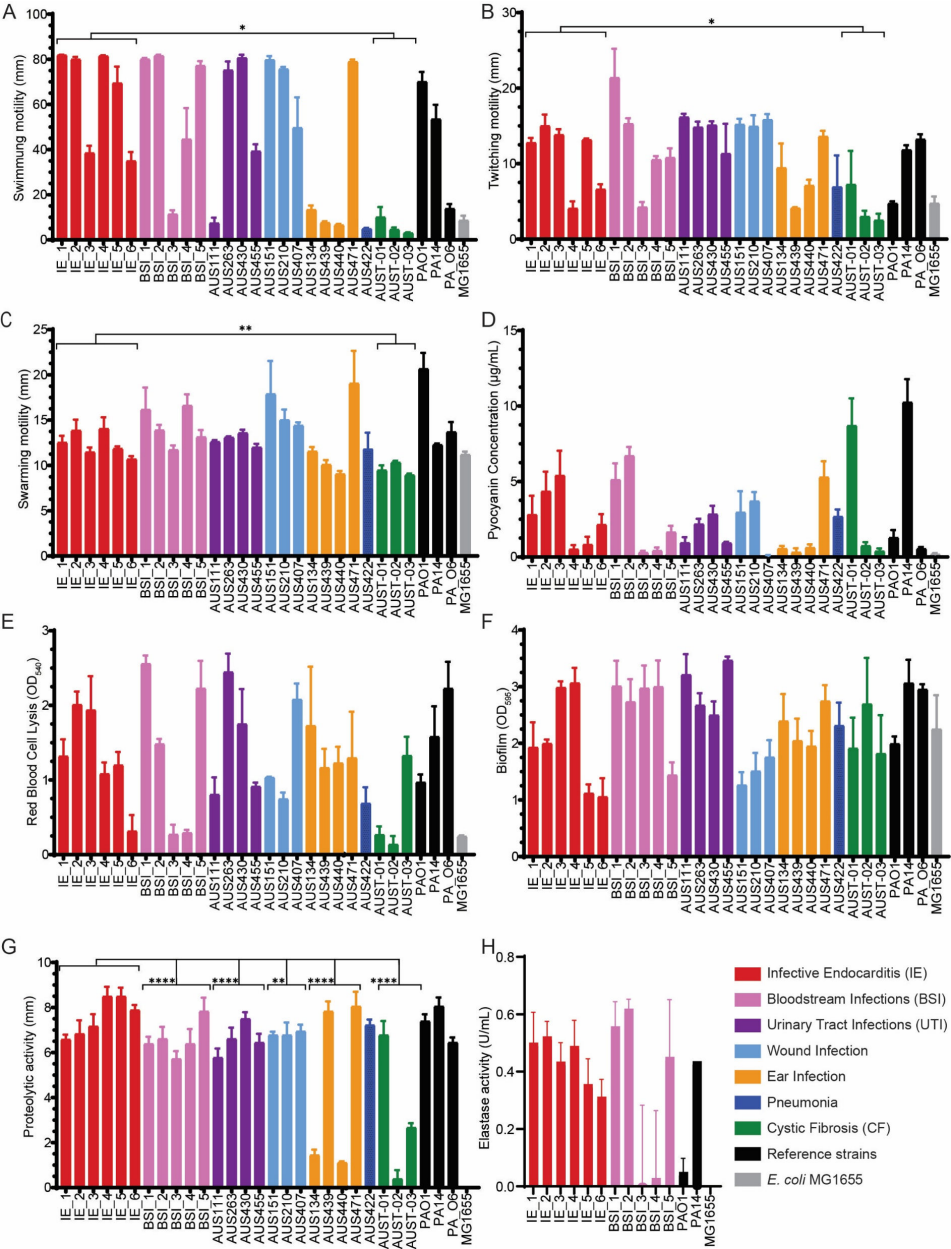
Phenotypic description of IE isolates in comparison to other clinical *P. aeruginosa* isolates from different infection types. As a reference, *E. coli* strain MG1655 was used. (**A**) Swimming, (**B**) twitching, and (**C**) swarming motility of the panel was quantified by distance traveled (mm) on appropriate motility agar after 24 h. (**D**) Pyocyanin concentration (µg/mL) in suspension. (**E**) Lysis of red blood cells (RBCs) was compared and quantified by OD_540_. (**F**) Biofilm formation was quantified by crystal violet staining and comparison of absorption (OD_595_). SEM, *n* = 3. (**G**) Proteolytic activity of the panel was defined by the digestion diameter (mm). (**H**) Elastase activity was determined using EnzChek Elastase Assay Kit after log-phase growth in lysogeny broth. Error bars indicate SEM, *n* = 3. Significance of IE isolates’ phenotypes was determined by unpaired Student’s *t*-test; *P < 0.05, ** <0.01, *** <0.001, and **** <0.0001.

The IE isolates were first examined for the motility phenotypes, including swimming, twitching, and swarming (Fig. 4A through C). In all three cases, the IE isolates had significantly (*P* < 0.05) greater motility than isolates from patients with CF. Swimming motility mean score was 58.27 (SD 31.95, 95% [34.46, 81.07]), twitching motility mean score was 6.564 (SD 5.27, 95% [0.9801, 12.45]), and swarming motility mean score was 58.27 (SD 2.01, 95% CI [35.46, 81.07]). Interestingly, proteomic analysis indicated reduced flagella protein levels, yet four of six IE isolates displayed swimming motility comparable to PAO1 and PA14, six of six IE isolates swarming motility comparable to PA14, along with upregulated type IV pili operon genes and high twitching motility similar to PA14.

One mechanism *P. aeruginosa* employs to control regulation is quorum sensing. Proteins under the regulation of quorum sensing and associated with *P. aeruginosa* virulence include pyocyanin production, elastase production, and biofilm formation (27). Here, we analyzed pyocyanin production (a key redox-active secondary metabolite toxic to host cells) across the panel of isolates. Pyocyanin production varied across the panel, but no infection group produced pyocyanin significantly different from another infection group (Fig. 4D). Reference strain PA14 was the highest producer (10.2 µg/mL), and in comparison to IE isolates, production was reduced. Among IE isolates, pyocyanin production varied, with minimal production in 2/6 isolates (<0.83 µg/mL).

Proteomics also showed the upregulation of proteins associated with heme oxidation and binding in IE isolates against PA14. Phenotypically, the majority of IE isolates, despite IE_6, were able to lyse red blood cells (RBCs; Fig. 4E). Isolates IE_2 and IE_3 demonstrated higher lysis activity than PA14, supporting proteomic findings that IE isolates upregulate proteins associated with RBC lysis and iron acquisition. Nutrient availability varies throughout the process of infection; for example, biofilm structures are nutrient limited (28). Panel isolates all produced biofilm; however, production was not significantly different when comparing infection groups (Fig. 4F). Among IE isolates, biofilm production was varied, with IE_5 and IE_6 demonstrating the lowest biofilm production across the cohort.

### IE isolates are highly proteolytic

Proteomic analysis identified significant upregulation of toxins, including proteases (LasA-B, PrpL, and Lap) and phenazines. Phenotypic assays suggested the relevance of toxin production in IE isolates due to their high proteolytic and hemolytic activity and production of pyocyanin (Fig. 4D and E), all factors associated with a virulent phenotype. We investigated non-specific proteolytic activity and found that IE isolates as a group had significantly higher proteolysis compared to other acute disease isolates from BSI (mean difference 0.9880, SD 1.2368, 95% CI [0.5264, 1.450]), UTI (mean difference 0.9936, SD 1.2010, 95% CI [0.5277, 1.459]), wound (mean difference 0.7436, SD 1.079, 95% CI [0.3279, 1.159]), ear infections (mean difference 2.966, SD 2.7703, 95% CI [1.757, 4.175]), and CF (mean difference 4.299, SD 2.7612, 95% CI [3.151, 5.447]; Fig. 4G). To more specifically investigate elastase activity (LasA and LasB associated), specific assays were performed with IE isolates and reference strains, PAO1 and PA14 (Fig. 4H). Elastase activity of IE isolates was significantly higher than BSI isolates (mean difference 0.2261, SD 0.2896, 95% CI [0.03419, 0.4179]) and in IE isolates in comparison to PAO1 activity (mean difference 0.4026, SD 0.3071, 95% CI [0.2509, 0.5543]); however, no significant difference was observed against PA14, which also had high elastase activity.

### Lipopolysaccharide and serum resistance of IE isolates

Lipopolysaccharide (LPS) is a key virulence determinant in many *P. aeruginosa* acute infections. To investigate LPS phenotype, O-antigen expression was visualized via western blotting, which showed that all IE isolates express smooth LPS, that is, expressed O-antigen (Fig. S2). Lipid A modifications can promote resistance to host immune response, and constitutive expression of these modifications is associated with chronic isolates such as from patients with CF (29). Fast lipid analysis technique (FLAT) identified that all IE isolates shared wild-type lipid A modification profiles after growth in lysogeny broth (LB) and blood (Table S4).

**Fig 5:**
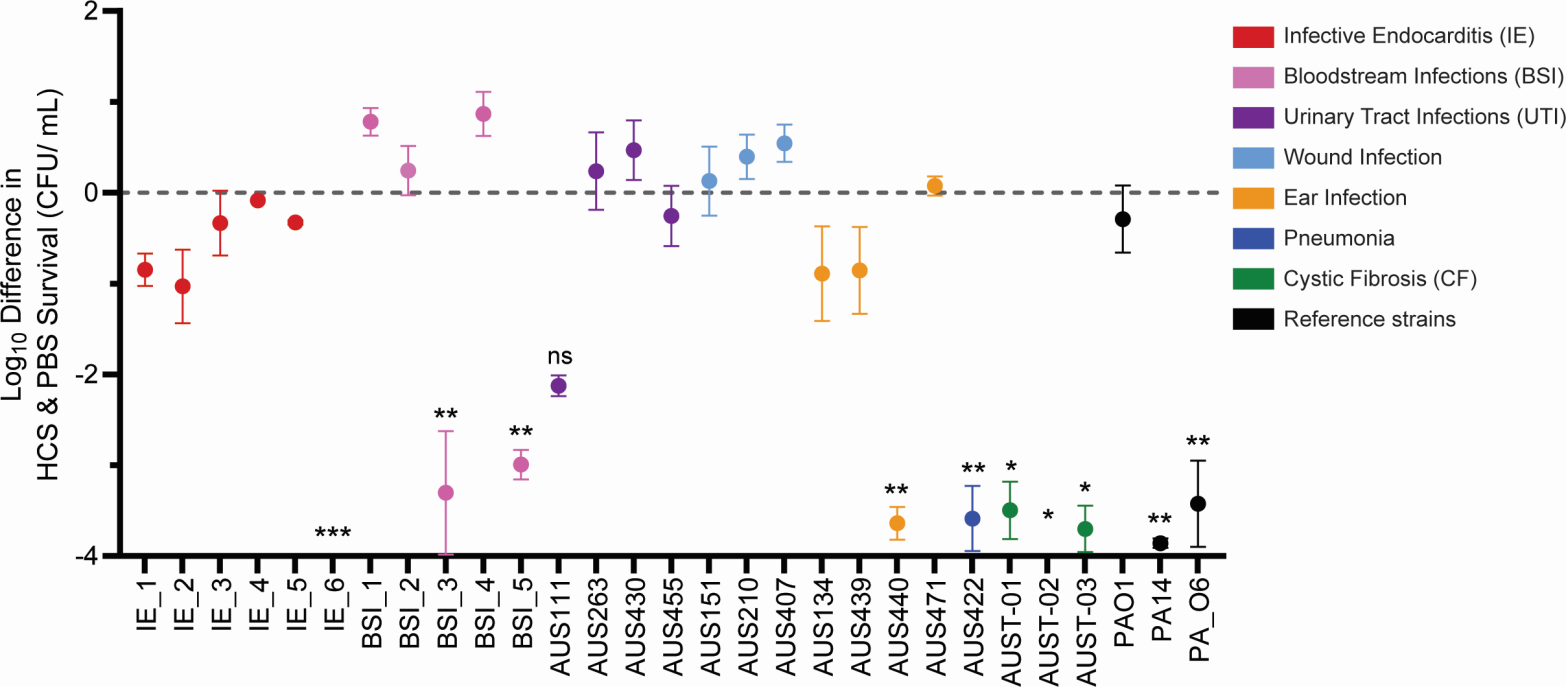
Complement-killing sensitivity of *P. aeruginosa* isolates from different infection types. Serum bactericidal assay was performed to determine the sensitivity of *P. aeruginosa* isolates to complement-killing. Bacterial killing in HCS (complement active) was measured as colony-forming units (CFU)/mL after 3 h incubation in HCS. Presented is the log-fold difference in bacterial survival in HCS compared to phosphate-buffered saline (PBS). Area under the curve (AUC) of HCS killing was compared to AUC of growth in PBS as a negative control. Significance of killing in HCS compared to PBS alone was determined by unpaired Student's *t*-test; *P < 0.05, ** <0.01, and *** <0.001. Error bars indicate SEM, *n* = 3.

Survival in the blood is important for bacteremia development, a common precursor for IE. Thus, resistance to serum-killing (i.e., complement-mediated killing) is an important trait. Thus, bacterial survival of the panel in healthy control serum (HCS) was investigated (Fig. 5). All but one IE isolate was resistant to serum killing. Overall, 65% of the panel was resistant to serum killing, with the majority of sensitive isolates from a chronic lung infection source. Overall, IE isolates were found to have virulence phenotypes similar to strains from other acute infections, with a notable increase in protease activity.

## DISCUSSION

Although *P. aeruginosa* remains a relatively rare cause of IE, it is associated with high mortality and recurrence rates, underscoring the urgent need for effective treatment strategies (9, 11, 30, 31). In this study, the genotypic, proteomic, and phenotypic profiles of *P. aeruginosa* isolates from IE cases across a hospital network in Brisbane, Australia, over a 20-year collection period were analyzed. While no unique molecular signature distinguished these isolates under the tested conditions, they exhibited virulence traits and phenotypes typically linked to acute infections. Proteomic analysis identified key virulence factors, including phenazines, proteases, and pili, which may be vital for disease establishment and persistence. Phenotypically, IE isolates all had high proteolytic activity, significantly higher than isolates from a range of other disease settings.

Both proteomic and phenotypic analyses revealed that proteases are upregulated in IE isolates in comparison to other *P. aeruginosa* disease isolates. Both host and bacterial proteases have been found to be vital in IE development and persistence. Proteomic analysis of staphylococcal and non-staphylococcal IE vegetations demonstrated extensive proteolysis within these structures from both bacterial and host proteins (32). Host proteolysis can promote infection suppression and targeted immune response to the site of lysis; however, when dysregulated, it can favor infection by modifying immune responses (e.g., degradation of immunoglobulins, complement, and cytokines) and contributing to vegetation growth (e.g., prevention of wound healing and extensive neutrophil recruitment) (32). In *P. aeruginosa*, bacterial proteases are well-characterized virulence factors in the context of other infections. For example, elastase or LasB has been associated with virulence in lung infections, with higher expression reported in early-stage infection of patients with CF (33, 34). Our results here strongly suggest that proteases contribute to *P. aeruginosa* virulence during IE, perhaps by contributing to cardiac damage, priming the surface for *P. aeruginosa* colonization and IE development, or contributing to surface barrier invasion and vegetation emboli generation for further infection dissemination, as previously described (32).

Proteomic analysis also revealed significant differences in protein expression between IE isolates and two reference strains. These findings highlight the adaptability of *P. aeruginosa* and suggest that specific regulatory mechanisms enable the pathogen to survive and proliferate in the cardiac environment. Notably, proteins involved in heme oxidation and heme-binding, such as PigA, Fhp, and NorC, were upregulated in IE isolates compared to PA14, potentially conferring a survival advantage in the bloodstream. One publication regarding *P. aeruginosa* IE pathogenesis characterized *pvdS*, encoding an oxygen-dependent sigma factor, as a potential IE virulence factor, supporting regulation of the infection environment as key (35). However, it is important to note that these strains were only compared to the reference isolates originally obtained from wound infections. Future studies that compare IE isolates to other infection types (such as BSIs) may better elucidate the factors required for the development of infective endocarditis.

Additionally, proteins associated with an acute infection phenotype were enriched, including pili involved in motility and adhesion, as well as quorum-sensing-regulated factors such as proteases, phenazines, and toxins. These factors are known to facilitate bacterial colonization and induce host cell damage, contributing to other *Pseudomonas* infections. Adhesion in the cardiac environment is challenged by high shear forces, necessitating strong attachment to surfaces. Adhesins play a critical role in *P. aeruginosa* pathogenesis and have also been implicated in the virulence of other IE-causing species. For example, in *S. aureus*, fibronectin binding protein A (FnBPA) is essential for colonization of heart valves (36, 37). Pilins of type IV pili, known to promote *P. aeruginosa* adhesion and surface sensing, were also upregulated in proteomic analysis. Specifically, PilY1, an adhesin located at the tip of type IV pili, was upregulated relative to PA14, while PilC and PilD were upregulated compared to PAO1. Phenotypic assays supported the importance of type IV pili, as the majority of IE isolates demonstrated high twitching motility. Additionally, quorum sensing does have a critical role in biofilm formation, coordinating the transition from planktonic to sessile growth and survival within the biofilm structure (28). As a species, *P. aeruginosa* is characterized as prolific at biofilm formation, causing increased difficulty in antimicrobial therapies. These findings align with the well-established model in which *P. aeruginosa* employs a coordinated suite of virulence factors to establish infection.

Proteomic analysis revealed that four proteins with proteolytic activity (LasA, LasB, PrpL, and Lap) were upregulated in the majority of IE isolates. In combination with other upregulated factors such as phenazines, these findings suggest that *P. aeruginosa* IE isolates may enhance host cell damage during infection to facilitate colonization and persistence. It is then suggested that, in synergy, pili, proteases, and phenazines contribute to increased pathogenicity in IE. However, functional validation using loss-of-function mutants and *in vivo* IE models is necessary to confirm their roles in disease progression.

Proteomics is limited by the detection of protein variants where sequences are too dissimilar, calling differential expression from reference strains. This is reflected by the apparent downregulation of some factors. For example, *P. aeruginosa* encodes 20 serotypes of LPS. Against reference strain PAO1, LPS-associated proteins (WbpABEI) were significantly downregulated in comparison to IE isolates. Similarly, *P. aeruginosa* encodes flagella variants (A and B types), and flagella-associated proteins (FliCDS) were downregulated against both reference strains.

There are two notable limitations of this work that should be taken into account when considering the outcomes. The first is the limited amount of *P. aeruginosa* IE strains available for study, leading to difficulty in having the statistical power to determine phenotypic differences between these strains in comparison to other *P. aeruginosa* infection isolates, and thus, many of the phenotypes are more descriptive in nature. Second, these strains were all isolated from the blood of patients with IE, not taken from the infection source itself. Thus, these single strains, which likely have been sloughed off from the biofilm, may not represent the biology or diversity of the colonizing bacteria. Both these limitations are due to the difficulty in obtaining *P. aeruginosa* IE isolates due to its comparative rarity and the acute nature of the infection. Despite this, the findings from this study are still significant as they represent the first comprehensive characterization of clinical isolates from patients with *P. aeruginosa* IE. Future studies will aim to validate these findings and further elucidate *P. aeruginosa* pathogenesis in IE.

The growing prevalence of antibiotic-resistant *P. aeruginosa* infections underlines the urgent need for novel therapeutic strategies (38, 39). This study focused on virulence factors due to their well-established roles in *P. aeruginosa* and other IE-causing bacterial species. Although no unique marker was identified among the IE isolates, phenotypic profiles most closely resembled the well-characterized PA14 strain, supporting both genomic and proteomic findings. This similarity suggests that IE-associated virulence may not rely on a single defining marker but rather rely on a combination of bacterial regulatory mechanisms, virulence expression, bloodstream access, and host susceptibility. A deeper understanding of *P. aeruginosa* virulence mechanisms is critical for identifying therapeutic targets and developing effective interventions for bacteremia and endocarditis.

## MATERIALS AND METHODS

### Bacterial strains

Six bacterial samples isolated from patients with *P. aeruginosa* endocarditis were collected from Royal Brisbane and Women’s Hospital for analysis (LNR/2019/QRBW/60,456). Five bloodstream isolates utilized were randomly selected from a 100-patient cohort of *P. aeruginosa* BSIs collected from three hospitals in Brisbane, Australia: Royal Brisbane Hospital, Princess Alexandra Hospital, and Prince Charles Hospital (HREC/2018/QRBW/49202) (40). The remaining *P. aeruginosa* panel isolates from people with cystic fibrosis, urinary tract infections, pneumonia, wounds, and ear infections were generously supplied by the International *Pseudomonas aeruginosa* Consortium (IPC) genome sequencing project as part of BioProject PRJNA325248 (41–44). In addition, three common reference strains that are well-characterized genomically and phenotypically were used: PAO1 (45), PA14 (46), and PAO6 (47). Metadata for strains are included in Table S2. Unless otherwise specified, the following methods were followed for culturing and storage of samples. *P. aeruginosa* was cultured in LB at 37°C while shaking (200 rpm).

### Genomic sequencing and analysis

Bacterial DNA was extracted from isolates for sequencing. QIAamp DNeasy Blood and Tissue kit was used to extract bacterial DNA from overnight cultures. The sample was prepared as per MicrobesNG guidelines for Oxford Nanopore Technologies next-generation sequencing. In brief, long reads were trimmed via Trimmomatic (v. 0.30) (48) and *de novo* assembled via SPAdes (v. 3.7) (49). Quality of sequence reads was determined by CheckM (v. 1.1.3) (50), and contamination <5% was accepted. Contigs were assembled into scaffolds against *P. aeruginosa* PAO1 reference sequence (NC_002516.2) using MAUVE (v. 20150226) (51). Genomes were annotated using Prokka (v. 1.14.5) (52) and Bakta (v. 1.7.0) (53). The sequence type of panel isolates was determined by plug-in of panel genomes to PubMLST database (https://pubmlst.org/) (54). Serotype of panel isolates was determined by plug-in of panel genomes to “Center of Genomic Epidemiology” database (PAst 1.0; https://cge.food.dtu.dk/services/PAst-1.0/) (55). A collection of virulence gene sequences was collected from reference strains as per as per McCarthy et al.(16). Nucleotide BLAST alignment identity scores are reported. To determine antibiotic gene presence, panel genomes were uploaded to “Comprehensive Antibiotic Resistance Database: Resistance Gene Identifier” (https://card.mcmaster.ca/analyze/rgi). Perfect and strict hits for antibiotic gene presence were collated. Genes that demonstrate differential presence and absence in the panel are represented.

### Proteomic analysis

Log-phase LB cultures (5 mL) were pelleted and washed twice in equal volume phosphate-buffered saline (PBS) (8,000 × *g*, 2 min, 4°C). Pellets were frozen in liquid nitrogen and processed by the Translational Research Institute proteomics core. Proteins were identified using the *P. aeruginosa* PeptideAtlas (56) reference database. Proteins that were significantly up- or down-regulated were determined via confirmed mass spectrometry peak and a significant (*P* < 0.05) abundance ratio against reference strains (PAO1 and PA14) in the majority (≥3) IE isolates. Fold change data excluded samples that had zero peaks for a protein. Data generated from proteomic analysis were determined to be of good quality and reliable (Fig. S1). Significantly regulated proteins against IE isolates are listed (Table S3).

### Antibiotic disk diffusion

Antibiotic susceptibility was determined via EUCAST standard for antibiotic disk diffusion (26). Briefly, bacterial colonies were resuspended in 1.5 mL 0.85% NaCl and standardized to a 0.5 McFarland turbidity standard. A sterile swab was dipped into the bacterial suspension and pressed against the tube side to remove excess liquid. Bacteria were evenly spread across Mueller-Hinton plates to achieve a lawn. Antibiotic disks were quickly applied to inoculated plates. Inoculated plates were incubated at 35°C for 20 h. The zone of clearance was measured and used to determine antibiotic sensitivity via EUCAST 2025 standards specific for *P. aeruginosa*.

### Sensitivity to serum killing

Killing of *P. aeruginosa* isolates in serum was determined via serum bactericidal assay as previously described (47). Briefly, HCS collected from the Australian Red Cross (2022/HE002409) was heat-inactivated at 56°C for 20 min. Bacterial cultures grown overnight were adjusted to OD_600_ 0.6, pelleted via centrifugation, and resuspended in PBS, creating a stock inoculum. A 50:50 mixture of HCS (active complement) and PBS (positive control) was mixed to create a 25 µL solution using a 1:10 diluted stock (2.5 µL). PBS alone with diluted stock was used as a negative control. Reactions were incubated at 37°C for 3 h. After incubation, the sample was serially diluted 10-fold in PBS, plated to LB agar, and incubated overnight at 37°C. CFU per milliliter was determined to calculate the fold change in bacterial growth as compared to the stock inoculum’s growth.

### LPS extraction and visualization

LPS extraction was performed as previously described (47). Extracted LPS was separated via sodium dodecyl sulfate-polyacrylamide gel electrophoresis (SDS-PAGE) and transferred to polyvinylidene fluoride membrane using iBlot2 Gel Transfer Device as per the manufacturer’s protocol. Membranes were incubated in casein (tris-buffered saline [TBS] and 5% non-fat milk powder) to block non-specific binding before incubation in *P. aeruginosa* polyvalent serotype-specific polysera 1:10,000 in dilution buffer (TBS, 0.1% Tween-20, 5% casein). Polyvalent group 1 (#213556, Denka Seiken) specificity to O-antigen serotype; O1, O3, O7, O8, O10, O12, O19, and polyvalent group 2 (#213563, Denka Seiken); O2, O5, O13, O14, O15, O16, O18, O20, and polyvalent group 3 (#213570, Denka Seiken); O4, O6, O9, O11, and O17. Membranes were incubated in secondary antibody, anti-rabbit IgG alkaline phosphatase-conjugated (A3687, Sigma-Aldrich). All incubation steps were performed for 1 h at room temperature with rocking, followed by four 5 min washes in TBST (TBS, 0.1% Tween-20). Membranes were developed with nitro-blue tetrazolium and 5-bromo-4-chloro-3’-indolyphosphate and imaged with BIO-RAD Image Lab Software.

### Biofilm microtiter plate assay

Biofilm formation was assessed as previously described (57) and compared between *P. aeruginosa* clinical isolates, reference *P. aeruginosa* strains (PAO1 and PA14), and *E. coli* strain MG1655. In brief, overnight cultures in LB were adjusted to an OD_600_ of 0.2. Using Corning 96-well round-bottom plate, 100 µL culture aliquot or media alone control was added to the corresponding wells. The plate was incubated at 37°C while shaking (200 rpm) for 18 h. Media were removed, and wells were washed twice with PBS. To stain biofilm, 150 µL 0.1% crystal violet was added to each well. The plate was incubated for 30 min at room temperature. Crystal violet was removed, and the plate was washed with water until blank media controls were clear. After the plate was dried, 150 µL ethanol:acetone (80:20) solution was added to each well to solubilize biofilms. The plate was incubated for 30 min while rocking slowly at room temperature. Absorbance was measured at 595 nm.

### Motility assays

Bacterial motility was quantified in two methods as previously described (58). The following was consistent between the two assays: overnight cultures were standardized to OD_600_ 1.0 and resuspended in PBS. Swimming motility: using a sterile pipette tip, the tip was dipped into bacterial suspension and was used to pierce 0.3% LB agar plate without touching the bottom of the petri dish. Twitching motility: using a sterile pipette tip, the tip was dipped into bacterial suspension and was used to pierce 1% LB agar plate, ensuring the tip touched the bottom of the petri dish. All inoculated LB plates were incubated at 37°C for 18 h. The diameter distance traveled by bacteria was measured to quantify bacterial motility.

### Proteolytic activity

Proteolytic activity was determined via quantification of digestion on LB agar + casein plates (1.5% non-fat milk powder) (58). Overnight cultures were standardized to OD_600_ 0.1 in PBS. A culture aliquot was plated in the center of agar. Inoculated plates were incubated at 37°C for 36 h. The zone of clearance was measured to quantify non-specific protein digestion. Elastase activity was determined as per EnzChek Elastase Assay Kit manufacturer’s protocol (E12056, Invitrogen). Briefly, log-phase culture supernatant was filtered (0.45 µM) and mixed with BODIPY-FL-labeled DQ elastin conjugate. Elastase activity (U/mL) was quantified against a positive control standard curve (diluted 1 U/mL porcine pancreatic elastase stock). Fluorescence readouts were measured using CLARIOstar Plus (λ_ex_ = 485 nm/λ_em_ = 535 nm) to determine the elastase activity of culture supernatant.

### Red blood cell lysis

Lysis of RBCs in suspension was investigated using Corning 96-well round-bottom plate as previously described (59). Overnight cultures were standardized to OD_600_ 0.1 in PBS. Defibrinated sheep blood (R54004, ThermoFisher Scientific) was centrifuged (4,000 × *g*, 10 min, 4°C) to separate RBCs. Red blood cells were washed in PBS three times and resuspended in cold RPMI-1640 (Gibco). RBCs were mixed with bacterial suspensions (100 µL, 1:1) in corresponding wells. Negative control: RBCs mixed with RPMI-1640 alone, positive control: RBCs mixed with 0.1% SDS. The plate was centrifuged at 1,500 × *g* for 10 min and incubated at 37°C for 1 h. The plate was centrifuged as before. Cell supernatant was transferred to a new plate, and absorbance was measured at 540 nm. The percentage of total lysis was calculated as follows: % = [(X − B)/(T − B)] ×100, where *B* (baseline) is the negative control absorbance, and *T* is the positive control absorbance.

### Pyocyanin production

Pyocyanin production was assessed as previously described (60). Briefly, isolates were grown in LB for 16 hr at 37°C shaking (200 rpm). Overnight cultures were standardized to OD_600_ 3.0. Cultures were pelleted, and supernatants were removed (300 µL). Supernatants were resuspended 1:1 in chloroform and centrifuged (8,000 × *g* for 2 min). Chloroform blue layer was added to 0.2 M HCl (3:1), and the mixture was briefly vortexed. The top layer was removed, and absorbance was measured at 520 nm. Pyocyanin concentration in culture supernatant (µg/mL) was calculated as follows: OD_520_ × 10 (dilution factor) × 17.72.

### Fast lipid analysis technique

Log-phase bacterial cultures in LB (5 mL) were pelleted and washed in PBS. Pellets were frozen in liquid nitrogen and lyophilized using VirTis SP Scientific BenchTop Pro with Omnitronics. Lipid A structural analysis was performed using FLAT as previously described (61). Briefly, freeze-dried pellets were resuspended in 50–100 µL of endotoxin-free water and briefly vortexed to combine. One microliter of the resuspended pellet was directly spotted onto a stainless steel 96-well MALDI target plate, followed by the addition of 1 µL of FLAT extraction solution (0.2 M anhydrous citric acid and 0.1 M trisodium citrate dihydrate). The FLAT target plate was incubated at 100°C in a humidified heat block for 30 min. Bacterial spots were washed with endotoxin-free water and air-dried, followed by the addition of norharmane matrix (10 mg/mL in 1:2 methanol:chloroform Sigma Aldrich, St. Louis, MO, USA). Mass spectra were collected in negative-ion mode using a Bruker microflex LRF within the 1,000–2,400 *m/z* range, 300 shots. Agilent ESI Tune Mix (Agilent, Santa Clara, CA, USA) was used as an external calibrant. MALDI-TOF MS data were processed and analyzed with flexAnalysis software (version 3.4).

## Data Availability

Genomic sequences of clinical endocarditis and bloodstream infection-derived isolates are available through NCBI BioProject PRJNA1211693 (see Table S2 for corresponding BioSample accession numbers). Remaining clinical *P. aeruginosa* isolates genome sequences are publicly available through NCBI BioProject PRJNA325248 (see Table S2). The following genome sequences of *P. aeruginosa* reference strains were used for comparison throughout this manuscript: PAO1 (GenBank: NC_002516.2) and PA14 (GenBank: NC_008463.1). Proteome data set is available through PeptideAtlas data set PASS05975.

## SUPPLEMENTAL MATERIAL

Supplemental tables and figures spectrum.03742-25-s0001.docx Tables S1−S4, and Figures S1−S2.

ASM does not own the copyrights to Supplemental Material that may be linked to, or accessed through, an article. The authors have granted ASM a non-exclusive, world-wide license to publish the Supplemental Material files. Please contact the corresponding author directly for reuse.
