# Supplementary material for: Genomic, proteolytic, and phenotypic characterization of Pseudomonas aeruginosa isolates causing infective endocarditis
Source: Microbiol Spectr. 2026 May 12;14(6):e03742-25. doi: 10.1128/spectrum.03742-25 (PMC13228066; doi:10.1128/spectrum.03742-25)
Supplement: Supplemental tables and figures — Tables S1−S4, and Figures S1−S2. [file spectrum.03742-25-s0001.docx]

**Supplementary Data**

**Supplementary Table 1.** Antibiotic resistance gene presence and absence in panel of P. aeruginosa isolates.

|  |  | IE | | | | | | BSI | | | | | UTI | | | | | Wound | | | Ear Infection | | | | P | CF | | | Reference | | |
| --- | --- | --- | --- | --- | --- | --- | --- | --- | --- | --- | --- | --- | --- | --- | --- | --- | --- | --- | --- | --- | --- | --- | --- | --- | --- | --- | --- | --- | --- | --- | --- |
|  |  | IE_1 | IE_2 | IE_3 | IE_4 | IE_5 | IE_6 | BSI_1 | BSI_2 | BSI_3 | BSI_4 | BSI_5 | | AUS111 | AUS263 | AUS430 | AUS455 | AUS151 | AUS210 | AUS407 | AUS134 | AUS439 | AUS440 | AUS471 | AUS422 | AUST-01 | AUST-02 | AUST-03 | PAO1 | PA14 | PA_O6 |
| Aminoglycoside resistance | APH(3')-IIb | + | + | + | + | + | + | + | + | + | + | + | | + | + | + | + | + | + | + | + | + | + | + | + | + | + | + | + | + | + |
|  | *pmpM* | + | + | + | + | + | + | + | + | + | + | + | | + | + | + | + | + | + | + | + | + | + | + | + | - | + | + | + | + | + |
| Fluoroquinolone resistance | *gyrA* | - | - | - | - | - | - | - | - | - | - | - | | - | - | - | - | - | - | - | - | - | + | - | + | + | - | + | - | - | - |
|  | *parE* | - | - | - | - | - | - | - | - | - | - | - | | - | - | - | - | - | - | - | - | - | - | - | - | - | + | - | - | - | - |
| Chloramphenicol / Fosfomycin/ Sulfonamide resistance | *sul1* | - | - | - | - | - | - | - | - | - | - | - | | - | - | - | - | - | - | - | - | - | - | - | - | - | + | - | - | - | - |
|  | *catB7* | + | + | + | + | + | + | + | + | - | + | + | | + | + | + | + | + | + | + | + | + | + | + | + | + | + | + | + | + | + |
|  | *fosA* | + | + | + | + | + | + | + | + | + | + | + | | + | + | + | + | + | + | + | + | + | + | + | + | + | + | + | + | - | + |
| Beta-lactamase resistance | *arnA* | + | + | + | + | + | + | + | + | + | + | + | | + | + | + | + | + | + | + | + | + | + | + | + | + | + | + | + | + | + |
|  | *pmrA* | + | + | + | + | + | + | + | + | - | - | - | | + | + | + | - | + | - | + | + | - | - | - | + | + | + | + | - | - | - |
|  | *basS* | + | + | + | + | + | + | + | + | + | + | + | | + | + | + | + | + | + | + | + | + | + | + | + | + | + | + | + | + | + |
|  | *cprR* | + | + | + | + | + | + | + | + | + | + | + | | + | + | + | + | + | + | + | + | + | + | + | + | + | + | + | + | + | + |
|  | *cprS* | + | + | + | + | + | + | + | + | + | + | + | | + | + | + | + | + | + | + | + | + | + | + | + | + | + | + | + | + | + |
|  | OXA-type | 486 | 494 | 494 | 396 | 50 | 486 | 494 | 847 | 488 | 906 | 50 | | 494 | 851 | 494 | 396 | 486 | 50 | 903 | 847 | 396 | 396 | 488 | 50 | 904 | 846 | 50 | 50 | 488 | 904 |
|  | PDC-type | 8 | 5 | 71 | 5 | 5 | 24 | 120 | 53 | 34 | 59 | 8 | | 24 | 3 | 407 | 8 | 3 | 8 | 8 | 53 | 5 | 5 | 124 | 3 | - | 3 | 3 | 1 | 34 | 117 |

**Abbreviations** IE; Infective Endocarditis, BSI; Bloodstream Infection, UTI; Urinary Tract Infection, CF; Cystic Fibrosis and P; Pneumonia

**Supplementary Table 2** Data availability of the panel of *P. aeruginosa* isolates utilized in this study.

| Sample ID | Strain | Source  (Host Disease State) | BioProject | BioSample | Location | Ref. |
| --- | --- | --- | --- | --- | --- | --- |
| IE_1 |  | Infective Endocarditis | PRJNA1211693 | SAMN46283490 | Brisbane, Australia | (43) |
| IE_2 |  | Infective Endocarditis | PRJNA1211693 | SAMN46283491 | Brisbane, Australia | (43) |
| IE_3 |  | Infective Endocarditis | PRJNA1211693 | SAMN46283492 | Brisbane, Australia | (43) |
| IE_4 |  | Infective Endocarditis | PRJNA1211693 | SAMN46283493 | Brisbane, Australia | (43) |
| IE_5 |  | Infective Endocarditis | PRJNA1211693 | SAMN46283494 | Brisbane, Australia | (43) |
| IE_6 |  | Infective Endocarditis | PRJNA1211693 | SAMN46283495 | Brisbane, Australia | (43) |
| BSI_1 |  | Bloodstream Infection | PRJNA1211693 | SAMN46283496 | Brisbane, Australia | (43) |
| BSI_2 |  | Bloodstream Infection | PRJNA1211693 | SAMN46283497 | Brisbane, Australia | (43) |
| BSI_3 |  | Bloodstream Infection | PRJNA1211693 | SAMN46283498 | Brisbane, Australia | (43) |
| BSI_4 |  | Bloodstream Infection | PRJNA1211693 | SAMN46283499 | Brisbane, Australia | (43) |
| BSI_5 |  | Bloodstream Infection | PRJNA1211693 | SAMN46283500 | Brisbane, Australia | (43) |
| AES-1R (AUST-01) |  | Cystic Fibrosis | PRNJA325248 | SAMN05226618 | Sydney, Australia | (44) |
| AUS23 (AUST-02) | 2D9A | Cystic Fibrosis | PRJNA325248 | SAMN05226616 | Brisbane, Australia | (44) |
| AUS52 (AUST-03) | EC22 | Cystic Fibrosis | PRJNA325248 | SAMN05226619 | Hobart, Australia | (44) |
| AUS111 |  | Urinary Tract Infection | PRJNA325248 | SAMN07423963 | Brisbane, Australia | (43) |
| AUS263 |  | Urinary Tract Infection | PRJNA325248 | SAMN07423985 | Brisbane, Australia | (43) |
| AUS430 |  | Urinary Tract Infection | PRJNA325248 | SAMN07423976 | Brisbane, Australia | (43) |
| AUS455 |  | Urinary Tract Infection | PRJNA325248 | SAMN10478456 | Brisbane, Australia | (41) |
| AUS151 |  | Wound | PRJNA325248 | SAMN07423972 | Brisbane, Australia | (43) |
| AUS210 |  | Wound | PRJNA325248 | SAMN07423919 | Brisbane, Australia | (43) |
| AUS407 |  | Wound | PRJNA325248 | SAMN07423932 | Brisbane, Australia | (43) |
| AUS134 |  | Ear Infection | PRJNA325248 | SAMN07423967 | Brisbane, Australia | (43) |
| AUS439 |  | Ear Infection | PRJNA325248 | SAMN07423993 | Brisbane, Australia | (43) |
| AUS440 |  | Ear Infection | PRJNA325248 | SAMN07423994 | Brisbane, Australia | (43) |
| AUS471 |  | Ear Infection | PRJNA325248 | SAMN07423936 | Brisbane, Australia | (43) |
| AUS422 |  | Pneumonia | PRJNA325248 | SAMN07423999 | Brisbane, Australia | (43) |
| PAO1 |  | Wound (Reference) | PRJNA331 | SAMN02603714 | Melbourne, Australia | (45) |
| PA14 |  | Burn Wound (Reference) | PRJNA386 | SAMN02603591 | Massachusetts, United States | (46) |
| PA_O6 |  | Keratitis (Reference) | PRJNA1211693 | SAMN46283490 | Newcastle, United Kingdom | (47) |

**Supplementary Table 3** Significantly regulated proteins in majority of IE isolates compared to reference strains (PAO1 and PA14). All proteins listed are significantly differentially expressed (p < 0.05, 2-fold change in abundance ratio) in ≥3 IE isolates against reference strains. Protein descriptions were sourced from pseudomonas.com.

| Protein Name | Locus Tag | Description |
| --- | --- | --- |
| AmiE | **PA3366** | Aliphatic amidase |
| AprD | **PA1246** | Alkaline protease secretion protein |
| AprE | **PA1247** | Alkaline protease secretion protein |
| AprF | **PA1248** | Alkaline protease secretion outer membrane protein |
| ArcD | **PA5170** | Arginine/ornithine antiporter |
| ArnA | **PA3554** |  |
| BauB | **PA0131** |  |
| BraB | **PA1590** | Branched chain amino acid transporter |
| Bfr | **PA4235** | Bacterial ferritin |
| CalB | **PA0366** |  |
| CbpD | **PA0852** | Chitin-binding protein CbpD precursor |
| CdsA | **PA3651** | Phosphatidate cytidylyltransferase |
| ClpS | **PA2621** |  |
| ClpV1 | **PA0090** |  |
| CobD | **PA1275** | Cobalamin biosynthetic protein |
| Der | **PA3799** | Conserved hypothetical protein |
| DppA3 | **PA4500** | Probable binding protein component of ABC transporter |
| FabZ | **PA3645** | (3R)-hydroxymyristoyl-[acyl carrier protein] dehydratase |
| Fhp | **PA2664** | Flavohemoprotein |
| FliC | **PA1092** | Flagellin type B |
| FliD | **PA1094** | Flagellar capping protein |
| FliS | **PA1095** |  |
| FolE1 | **PA3438** | GTP cyclohydrolase I precursor |
| FpvA | **PA2398** | Ferripyoverdine receptor |
| GcvH1 | **PA2446** | Glycine cleavage system protein H2 |
| GcvP2 | **PA2445** | Glycine cleavage system protein P2 |
| GlyA2 | **PA2444** | Serine hydroxymethyltransferase |
| Hcp | **PA0085** |  |
| HisF2 | **PA3151** | Imidazoleglycerol-phosphate synthase, cyclase subunit |
| HisH2 | **PA3152** | Glutamine amidotransferase |
| HutU | **PA5100** | Urocanase |
| Imm2 | **PA1151** | Pyocin S2 immunity protein |
| KatE | **PA2147** | Catalase HPII |
| KdpB | **PA1634** | Potassium-transporting ATPase, B chain |
| Lap | **PA2939** |  |
| LasA | **PA1871** | Protease precursor |
| LasB | **PA3724** | Elastase |
| LecA | **PA2570** |  |
| MmsA | **PA3570** | Methylmalonate-semialdehyde dehydrogenase |
| MmsB | **PA3569** | 3-hydroxyisobutyrate dehydrogenase |
| ModA | **PA1863** | Molybdate-binding periplasmic protein precursor |
| Ndk | **PA3807** | Nucleoside diphosphate kinase |
| NirS | **PA0519** | Nitrite reductase precursor |
| NorC | **PA0523** | Nitric-oxide reductase subunit C |
| NqrB | **PA2998** | Na+-translocating NADH:ubiquinone oxidoreductase subunit Nrq2 |
| OprD | **PA0958** | Basic amino acid, basic peptide and imipenem outer membrane porin precursor |
| PchB | **PA4230** | Salicylate biosynthesis protein |
| PchE | **PA4226** | Dihydroaeruginoic acid synthetase |
| PchF | **PA4225** | Pyochelin synthetase |
| PhzA2 | **PA1899** | Probable phenazine biosynthesis protein |
| PhzB1 | **PA4211** | Probable phenazine biosynthesis protein |
| PhzB2 | **PA1900** | Probable phenazine biosynthesis protein |
| PhzD1 | **PA4213** | Phenazine biosynthesis protein |
| PhzF1 | **PA4215** | Probable phenazine biosynthesis protein |
| PhzM | **PA4209** | Probable phenazine-specific methyltransferase |
| PhzS | **PA4217** | Flavin-containing monooxygenase |
| PigA | **PA0672** | Heme oxygenase |
| PilA | **PA4525** | Type 4 fimbrial precursor |
| PilC | **PA4527** | Type IV pilus assembly protein |
| PilD | **PA4528** | Type 4 prepilin peptidase |
| PilY1 | **PA4554** | Type 4 fimbrial biogenesis protein |
| PncB1 | **PA4919** | Nicotinate phosphoribosyltransferase |
| PqsB | **PA0997** | Secondary metabolite biosynthetic process |
| PqsC | **PA0998** |  |
| PqsE | **PA1000** | Quinolone signal response protein |
| PrpL | **PA4175** | Protease IV |
| Pth | **PA4672** | Peptidyl-tRNA hydrolase |
| PxpA2 | **PA2112** | Conserved hypothetical protein |
| RhlA | **PA3479** | Rhamnosyltransferase chain A |
| RplR | **PA4247** | 50S ribosomal protein L18 |
| RplT | **PA2741** | 50S ribosomal protein L20 |
| RpmA | **PA4567** | 50S ribosomal protein L27 |
| RpmD | **PA4245** | 50S ribosomal protein L30 |
| RpmG | **PA5315** | 50S ribosomal protein L33 |
| RpmH | **PA5570** | 50S ribosomal protein L34 |
| RpmJ | **PA4242** | 50S ribosomal protein L36 |
| RpsL | **PA4268** | 30S ribosomal protein S12 |
| RpsN | **PA4250** | 30S ribosomal protein S14 |
| RpsR | **PA4934** | 30S ribosomal protein S18 |
| SpeH | **PA4773** |  |
| SsuD | **PA3444** | Conserved hypothetical protein |
| TreA | **PA2416** | Periplasmic trehalase precursor |
| TrpG | **PA0649** | Anthranilate synthase component II |
| TssC1 | **PA0084** |  |
| UreG | **PA4893** | Urease accessory protein |
| WbpA | **PA3159** | UDP-N-acetyl-d-glucosamine 6-Dehydrogenase |
| WbpB | **PA3158** | UDP-2-acetamido-2-deoxy-d-glucuronic acid 3-dehydrogenase |
| WbpE | **PA3155** | UDP-2-acetamido-2-dideoxy-d-ribo-hex-3-uluronic acid transaminase |
| WbpI | **PA3148** | UDP-N-acetylglucosamine 2-epimerase |
| YciB | **PA3201** | Conserved hypothetical protein |
|  | **PA0034** | Probable two-component response regulator |
|  | **PA0041** | Probable hemagglutinin |
|  | **PA0404** | Conserved hypothetical protein |
|  | **PA1195** | Dimethylarginine dimethylaminohydrolase |
|  | **PA1545** | Hypothetical protein |
|  | **PA2116** | Conserved hypothetical protein |
|  | **PA2567** | Hypothetical protein |
|  | **PA3240** | Conserved hypothetical protein |
|  | **PA3430** | Probable aldolase |
|  | **PA4299** |  |
|  | **PA4478** | Conserved hypothetical protein |
|  | **PA4738** |  |
|  | **PA5481** | Hypothetical protein |

Supplementary Table 4 Lipid A mass spectrometry peaks. Lipid A modifications were characterized after fast lipid analysis technique (FLAT) analysis and presence or absence of spectrum peaks (m/z) are classified.

| Strain | *m/z* 1275 | *m/z* 1291 | *m/z* 1366 | *m/z* 1381 | *m/z* 1446 | *m/z* 1462 | *m/z* 1526 | *m/z* 1542 | *m/z* 1616 | *m/z* 1632 | *m/z* 1684 | *m/z* 1700 |  | Key | |
| --- | --- | --- | --- | --- | --- | --- | --- | --- | --- | --- | --- | --- | --- | --- | --- |
| IE_1 | + | - | + | - | + | + | + | - | + | - | + | - |  | + | Peak observed |
| IE_2 | + | - | + | - | + | + | + | - | + | - | + | - |  | - | No peak observed |
| IE_3 | - | + | - | + | + | + | - | + | - | + | - | + |  |  |  |
| IE_4 | - | + | - | - | + | + | + | - | + | - | + | - |  |  |  |
| IE_5 | + | - | - | - | + | + | + | - | + | - | - | - |  |  |  |
| IE_6 | + | - | - | - | + | + | + | - | + | - | + | - |  |  |  |

**Supplementary Figure 1** **Proteome data after growth in nutrient-rich media (LB).** Protein expression of infective endocarditis isolates (IE) was compared to expression of PAO1 and PA14 reference strains. Abundance ratios, indicating fold-change in protein expression against reference strains, of significantly regulated proteins are plotted against A) PAO1 and B) PA14.


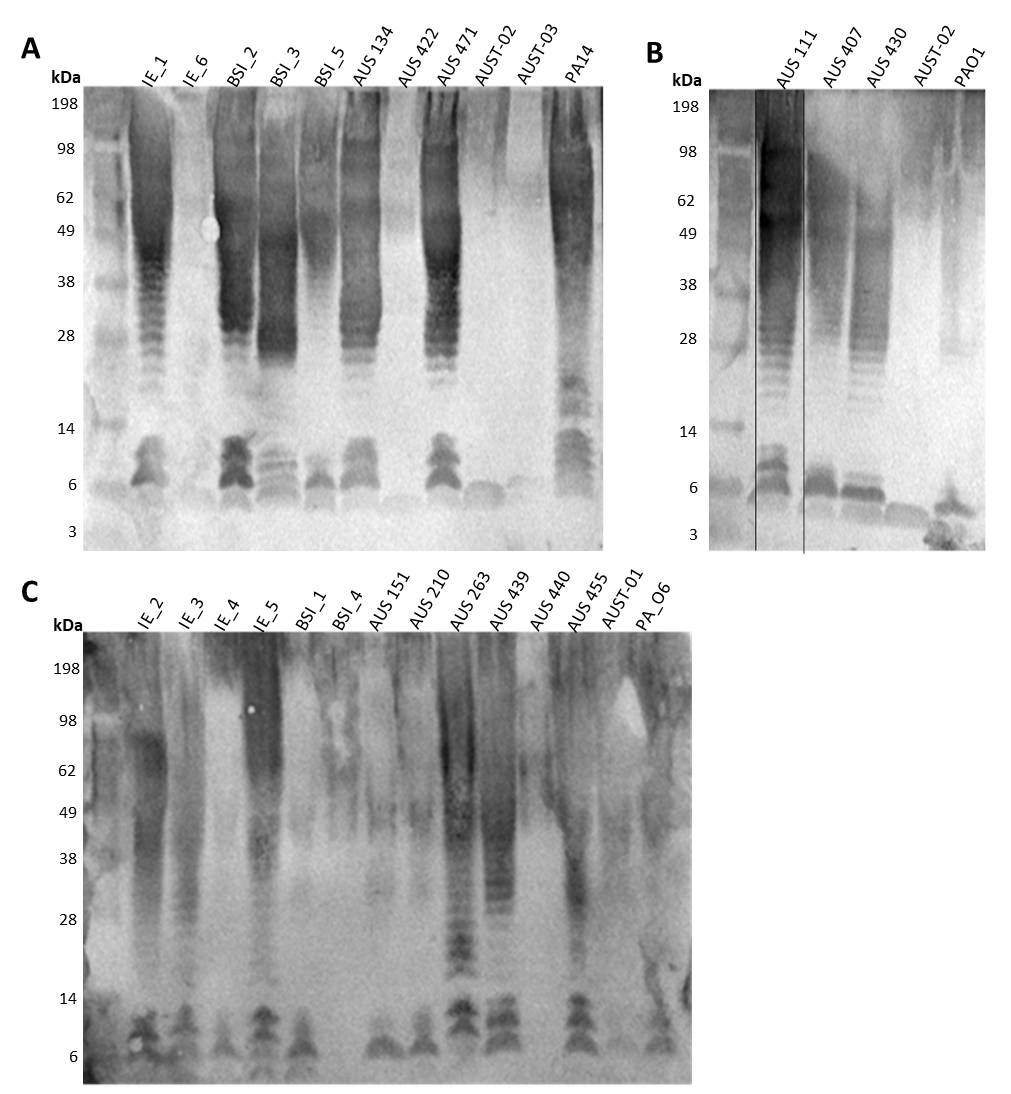


**Supplementary Figure 2** **Lipopolysaccharide (LPS) presentation of the panel of P. aeruginosa isolates.** LPS was extracted and separated via SDS-PAGE, visualized via western-blotting. Specific anti-O-antigen polysera was used to visualize and LPS presentation of the panel of isolates. O-antigen specific primers were used to visualize serotype-specific isolates as follows; A) Serotypes screened; O1, O3, O7, O8, O10, O12, O19, B) O2, O5, O13, O14, O15, O16, O18, O20, and C) O4, O6, O9, O11, O17.
